# Selections and Abstracts

**Published:** 1900-01

**Authors:** 


### *SELECTIONS AND ABSTRACTS.*

### THE MEDICAL ASPECT OF LIFE INSURANCE.*

Life insurance stands prominently forward as one of the institutions of modern life that is eminently unselfish. We know that its object is the protection of those individuals whom death has deprived of their support, and that it entails self-denial and forethought on the part of the insured to meet his premiums, which fact, in itself, reacts upon him advantageously by engendering thrift and economy, and fostering the best feelings of our common humanity. It has been noted that the more the principles of life insurance are understood, the more certain are they to be appreciated and acted upon; and while they give to society a guarantee for the uprightness and honesty of the individual, he in return, assists in rendering more firm and stable the groundwork of the Republic; for the number of policy-holders in a given locality may be taken as a fair index of the prosperity of that community, as they afford direct evidence of the existence of those qualities—thrift, forethought and consideration of others—upon which our social comfort and happiness chiefly depends.

While not an American institution in the sense of having had

*By Dr. S. Oakley Vanderpoel. (Medical Director New York Life Insurance Co.)

its origin in this country, life insurance, as at present carried on, is peculiarly American, in that it here first became an important and necessary factor of our civilization. Carried on for many years in England and on the Continent in the form of small, private enterprises, satisfied with extending its benefits to those who sought them out, it remained for American energy to develop the institution into one of the grandest benefactions which has marked the progress of human society.

Life insurance, while largely an economic and financial system, has an important medical aspect, and is carried on with the cooperation of our profession; in one form or another it enlists a considerable proportion of the energies of our fellow-practitioners. Most of you have probably, at one time in your lives, made examinations for insurance and many of you, I doubt not, now gain from similar work, a welcome addition to your professional incomes; and it is because of the peculiarly close relation of our profession to the business of life insurance that I feel justified in taking up your time with a brief consideration of a few of the many questions which that business involves. I think that the first impression of one unacquainted with medical work in life insurance—and I confess that I at one time shared in it—is that the physician well equipped in his profession should be able to conduct life insurance examinations, or to fill any of the functions of the life insurance expert, without any preliminary training. Very soon after taking up the work the practitioner becomes undeceived on that point; for not only is it necessary to become acquainted with the many details in which the various life and industrial offices differ among themselves, but speaking broadly and with reference to a matter of much more vital importance, it is necessary, it seems to me, for the physician to completely change his point of view with regard to the person whom he examines.

When examining for life insurance, most physicians who have not had much experience in such matters are very apt to begin their work with the idea that they are going to make a diagnosis—the whole mental attitude is that of the clinician who studies his case with a view to finding out in how many different organs the case under observation is unsound, or wherein the functions are not properly performed. This is a very commendable task for any physician to set himself to do, and he is indeed a capable man who is able to accomplish it. For purposes of life insurance work, however, this mental attitude seems to me to be wrong; by this I do not mean that a diagnosis is unnecessary, or that facts should not be taken into consideration which have a bearing upon the central question involved. What I do mean is that the examiner should always remember that the central question is not—what is the matter with this man; but rather—how long will this man probably live?

The keen horseman looks at the horse in the paddock before the race, not with the eye of the trained veterinarian, but rather with a view to measuring his capacity to resist the exhausting strain of the race. In arriving at his conclusions he takes into account the horse's previous record, his pedigree, the care he has received in training, and so on; but the central factor in his mind is the condition of the vitality of the horse. It is just in this way that the medical examiner should measure the capacity of the applicant to live; and this is what I meant when I said that the practitioner when he examines for insurance must change his point of view. As our knowledge of the significance of other factors which enter into the value of a risk increases, we shall undoubtedly be able to estimate lives much more closely than we do at present; but this one of vital capacity, of ability to live, must always remain for the local examiner to decide; and he is the best examiner who learns to judge his subject most accurately from this point of view.

The medical aspect of life insurance, like other branches of medicine, has not stood still in the past ten years; not only along the business lines, but also along the medical, has progress been made. We, as medical men, have from time to time wondered at the action taken by insurance companies in declining risks with whose physical condition we, as physicians, were personally conversant, knowing as we did that the lives were insurable, even if they did possess some physical impairment, notwithstanding which, in our opinion, they had a tenure of life that should not have excluded them from the benefits of life insurance. How many cases have come to our knowledge of applicants for insurance who have first been informed of the presence of a cardiac murmur when they submitted themselves to the life insurance examiner, and which, in consequence of the findings of the examiner, the company has seen fit to decline. The applicant has always considered himself a perfectly healthy man, has never experienced any of the subjective symptoms of cardiac disease, and consequently is greatly disturbed by the result of the examination. He then submits himself to his regular attendant, who, after a careful examination of his chest, informs him that there is a slight murmur, perhaps irregularity of his cardiac action; but assures him that in his (physician's) opinion it is of little or no moment, and will not materially shorten his life.

This picture, as I say, is so familiar to us all that we are quite prepared with our answer to an applicant when one of our patients informs us that he has been declined insurance in consequence of heart disease, albuminuria or some kindred ailment.

It seems to us, therefore, as medical men, that life insurance companies in the past have been too rigid and stringent in their rulings. The reason for this conservative action on the companies' part is not difficult to find, for they (the companies) likewise recognize the fact that certain cases of heart disease do have a considerable longevity, and therefore ought to be insurable; but as the data upon which life tables have been calculated have usually been made up from selected lives, those in which an impairment occurs are necessarily to be excluded—a case must be considered as one of a class, and not as an individual.

This other side of the picture has shown itself to me in the past six years, during which time I have been associated with one of the larger insurance companies engaged in underwriting lives. When I first became interested in life insurance, this question of damaged, or sub-standard lives—as they are termed in insurance parlance—made a decided impression upon my mind, because these men as a rule are persons who really require insurance, and it seemed, if sufficient data could be obtained—for instance, on cases of cardiac disease—proper actuarial calculations could be made which would permit it.

The axiom which was enunciated by Mr. Babbage years ago regarding the insurance of selected lives is equally applicable to the sub-standard or under-average lives. He said: "Nothing is more uncertain than the duration of life when the axiom is applied to the individual; but there are few things less subject to fluctuation than the duration of human life in a multitude of individuals." Hence, all that is necessary for us to do as insurance men is to acquire sufficiently large groups of given impairments upon which calculations can be based.

This subject, namely, how to assess equally the value of under-average lives, has attracted the attention of insurance medical directors on the other side, as the late address of Dr. Muirhead before the British Medical Association at Edinburgh would indicate. So far as we are able to ascertain from Dr. Muirhead's address, no systematic attempt has been made to collect and differentiate the various impairments, for he states he has no fixed and mathematically correct data by which to guide his decisions; they have no "aggregate groups of persons suffering from the same disorder, though perhaps differing greatly in form, which might supply a basis by which to adjudicate upon the special case before him; and if the extra rate recommended by the medical officers has in the past saved the company from loss, he is bound to admit that this happy result has been arrived at without the aid of any scientific formula, and by merely empirical methods.

The remedy suggested by Dr. Muirhead for this state of things is, to gather data sufficient to guide physicians in their estimate of extra risks, and to place them in a position to supply the actuaries with statistics upon which to calculate how much the acceptance of lives of unhealthy men is diminished in special groups of disease.

In the company with which I am connected, my confrère, Dr. O. H. Rogers, has for the past six years been engaged upon this vast subject, and with the immense amount of material at hand has separated these impaired lives into groups, and from these deduced his calculations. As yet his work is not sufficiently complete to permit of publication, but before long he hopes to present this matter to the profession, and it will be the first and most extensive work on this topic which has yet been submitted. You can readily appreciate the difficulties which have beset him in classifying the many thousands of damaged lives. Take, for instance, his study of tuberculosis; it was first intended to limit the study of consumption in the family record to those cases which presented absolutely no other defect; but it was soon found that, with our present knowledge of what constitutes impairment, it would be exceedingly difficult to draw the line between cases presenting only the impairment of a consumptive family record and those which present some other impairment as well. It was therefore decided to place in this group all cases presenting a family history of consumption, and the records consequently represent a slightly worse mortality than if the impairment was simply one of consumptive taint. This error, however, tends to offset the error just referred to, and gives weight to the conclusion that the results here arrived at are not far from correct. His search resulted in the accumulation of about 10,000 cases, of which 3,000 referred to policies not placed, the policies being applied for during a period of 15 years, and if kept in force carried for a maximum period of 25 years and a minimum period of 10 years. Almost all of these are of different lives, but the same life when insured at different times has been treated as so many separate lives.

In the accumulation of statistics he has pursued a somewhat different method from that formerly in vogue—a study of the death records of a company. It seemed that if it were possible to study applications on all policies, say between the years 1870 and 1884 inclusive, no matter whether they were accepted or rejected by the company, and if these were reviewed and an abstract made of each case presenting a family history of consumption, a much more accurate idea of the impairment could be obtained than from a study of the death losses of the company alone. The life history of each case was then made out, and then the fact noted whether the risk was still in force; and, if not, the mode of termination, whether by lapse, purchase, death or maturing policy, was recorded—the record being made as of December 31, 1898.

As a result of these observations, which have included all the other forms of impairments, such as rheumatism, albuminuria, albuminuria associated with Bright's, cardiac, glycosuria, simple excessive overweight and underweight,—I say, as a result of these, it has been possible to offer insurance upon a scientific basis to proponents who formerly were denied the benefits to be derived from it. Therefore we are in a position to instruct our medical examiners throughout the country to the effect that we are now able so to measure these impaired lives as to offer them some desirable form of insurance, and, in consequence, it becomes necessary for them, in examining such cases hereafter, to give us the fullest possible details of any impairment, whatever its character or degree, so that we may have before us data on which to base an intelligent estimate of the insurance value of the risk, and they should therefore submit the risk subject to the unfavorable features thus fully described by themselves.

If, for instance, it be a mitral murmur, we desire to know first, whether it is systolic or diastolic, the position of the apex, the compensation and the character and frequency of the pulse; the examiner then submits the risk subject to the aforesaid findings. This relieves the examiner entirely from the responsibility of accepting or rejecting an individual, and places that responsibility where it belongs—upon the Home Office of the company.

Another matter of importance to which I should like to ask your attention for a moment is that of the peculiar difference of mental attitude between the patient and the applicant for insurance. When he consults you, your patient gives you a full and frank history of his case; he endeavors to lay all the facts before you; he comes to get your advice and assistance, and his whole mental attitude is without reserve, and is one of cooperation.

When he is a candidate for life insurance the case is very different; his memory for details is less accurate, he forgets facts which, as a patient, he would be likely to remember; his state of mind is one of antagonism. I do not mean by this that most persons, when they are examined for insurance, give what they know to be anything less than the facts; I do not believe this is the case. A person presents himself for examination with the idea that he is a good risk, and his bias of mind in that direction is so strong as to color (I doubt not unconsciously) much that he tells you. On this account a medical history for life insurance is a very different matter from that which you will obtain from your patient. It requires time for any physician to learn to adjust himself to this very dissimilar mental attitude. The skilled medical examiner has learned this lesson and practices it.

Life insurance is a question of large numbers of lives, and the examiner should cultivate the habit of mind which expresses itself in terms of lives. He should ask himself,—if I had 1,000 such persons as this I am now engaged in examining, would they probably live as long as a similar number of other healthy lives? Or, if not a good life, how much more rapidly will 1,000 persons like this I am now examining die as compared with 1,000 healthy lives?

The first of these questions places before the examiner the problem which he is expected to solve by his study of the case. The second, while formerly quite unnecessary, is now being urged upon those who work in this branch of medicine, by reason of the tendency among life insurance companies to broaden the field of their beneficence so as to extend the benefits of insurance to those persons who, in consequence of some impairment, have heretofore been regarded as quite uninsurable. In England it has been the practice for many years to offer insurance at special rates to persons of unsound health, not seriously diseased, but impaired by reason of valvular lesion of the heart, albuminuria, asthma, gout, and so on; but with us, until very recently, all these conditions have been regarded as either bars to insurance or as requiring the limitation of the insurance to very short periods of time on endowment plans. Now, however, since the work has been taken up systematically of offering insurance to these impaired lives, I do not doubt that very soon American energy will again push us far in advance of our transatlantic cousins. This brings up several questions which have been the subject in time past of deliberation by this society, such as: Should life insurance companies extend the benefits of insurance to persons whose urine contains albumen, *i. e.*, the so-called cyclic albuminuria? Or, should they disregard some types of heart murmur or low grades of emphysema or true spasmodic asthma? It is not my purpose to occupy your time to-night with a consideration of such questions as these; they may well serve as profitable subjects for some of our future meetings. Behind them all, however, lies a fact which is vital to all of them, and to which I may briefly refer in closing without imposing too much upon you indulgence.

The success of a life insurance company is a question of careful management, and includes not only the questions of expense rate and of interest rate, but also the question of death-rate, and this factor of death-rate needs to be guarded just as carefully as that of interest rate or of expense of management.

I think we shall have a better idea of this mortality element if we assume, as a concrete example, that the membership of a company is made up entirely of persons at age thirty; the ratio of deaths to the total membership in this imaginary company during the first year would be only 8.43 per 1,000; during the second year, only 8.51 per 1,000; during the tenth year, 9.79 per 1,000; during the fifteenth year, 11.16 per 1,000. Now, let us suppose that we introduce into the membership of this company cases not quite up to the standard, and that on account of their various impairments three additional deaths per 1,000 occur in the first year, and and that this excess of mortality is continued year after year, so that in the fifteenth year it amounts to an addition of four deaths per 1,000. Surely this increased mortality seems to us very slight compared with the mortalities we are accustomed to in our studies as medical practitioners; and yet the addition to the death-rate from year to year of these numbers increases the mortality by thirty-three per cent.—an increase which, in the long run, would seriously disturb the stability of any life company. Indeed, the membership in any life company must be made up of lives among which the mortality will not exceed the expected rate, not only during the earlier years of their membership, but also during the later years.

This brief explanation will suffice, I think, to show why life companies are conservative in the matter of insuring persons with albuminuria, heart murmurs and other similar impairments. It may be perfectly true that cyclic albuminuria, some favorable forms of heart disease and other similar impairments, show very little additional mortality in the earlier years. Granting that this is true, it is only necessary that they should show a slight additional mortality to make them very doubtful risks; and which of us is prepared to affirm that, as they advance in years, this excess of mortality will not increase.

Looked at from this point of view, I am inclined to believe that many of you would hesitate to affirm that such persons are normal lives. In the face of this test of years of exposure, the criticism so often heard that life insurance companies are not justified in rejecting albuminuria falls to the ground.—*Canada Medical Record.*

---

### THE DIAGNOSIS OF THE PRE-TUBERCULOUS STAGE.

In the amount of attention which at the present day is being devoted to the study of tuberculosis, no excuse need be offered for bringing its salient features constantly before the reading medical fraternity, even though nothing new may be added to the subject, and recognizing that the medical mind is perhaps gorged to satiety. When we have repeatedly thrust upon us each and every month by the provincial sanitary authorities the fact that the death-rate in this province of barely two million souls approximates two hundred lives per month, and that in all probability 300,000 of that population is destined to succumb to one form or another of the disease, the vast majority from pulmonary tuberculosis; when we all acknowledge and recognize that given cases where the disease exists in the pulmonary organs before the appearance of sputum or the demonstration of the specific tubercle in apparent sputum; when it now cannot be successfully gainsaid that there is a very large percentage of permanent cures in such cases, under properly regulated sanitoria treatment; surely, then, it cannot be too strongly forced home upon the mind of the general practitioner the necessity of fortifying himself with every possible knowledge to this end, that he may be as near perfect as it is possible to be in diagnosing the existence of phthisis pulmonalis in this ante-tuberculous period.

The advancing tide of scientific thought and research has no doubt long ago compelled Von Ziemssen to renounce the doctrine enunciated by him some five or six years after the discovery of the bacillus by Koch—"No tubercle in the sputum, no pulmonary tuberculosis."

The most brilliant diagnosticians the world over now hold to the opinion that the tubercle bacilli may remain quiescent for many months, almost a year, without outward manifestation before their demonstration in the phthisical sputum; therefore it can readily be conceived how superlatively important it is to be able to diagnose the existence of the disease before the appearance of the bacilli in the sputum.

There are, moreover, pulmonary complaints of bronchitic and asthmatic individuals, the subjects of periodic attacks of these, which in the intervals will defy detection at the hands of the most astute diagnostician, especially in those persons who have something to gain by concealing their true condition.

The difficulty of diagnosing pulmonary tuberculosis in this early stage is well illustrated in Turban's statistics where, out of 408 patients received 2.7 per cent. only came with the disease in this first stage, two of these beginning under his own observation.

Frequent and regular estimation of the temperature every two hours, from 8 A.M. to 10 P.M., is one most important particular which ought to be strictly carried out and never omitted; and the menstrual period in women is a time when the existence of tubercle may betray itself by this method. In all cases Clifford Allbutt thinks that it will be sufficient to take this by the mouth, first ascertaining that no hot or cold drinks have just been taken and the face not recently exposed to an external cold draught. The thermometer should be left there from five to ten minutes.

Within the last decade the great advances in the science of pathology has taught us where to look for these first lesions, *i. e.*, directly over the posterior apical and subapical areas. Osler tells us that these lesions are not, as a rule, at the extreme apex, but generally about an inch or an inch and a half below it, and located postero-externally. The importance of sounding, then, in the supra-spinous fossæ, is indicated.

Emphasis ought to be placed on the physical examination of the apex of the lower lobe of the right lung, posteriorly, corresponding to a spot opposite the fifth dorsal spine. Here the arm should be so placed that the hand rests on the opposite shoulder, with the elbow horizontal, so as to lessen the thickness of the thoracic wall in that region. The patient should be requested to give a few coughs, after which the stethoscope should be placed over the spot and any adventitious sounds noted. Of course, it goes without saying, that every patient ought to be stripped to the waist and examined in a clear light. Dr. Allbutt places very little importance on jerky breathing, but emphasizes any lagging in the expansion of either apex with harshness of the murmur and loss of the vesicular quality. Although moist râles are probably absent in the great majority of these cases, yet if a single mucous click is heard, that clinches the diagnosis. As for percussion in these early conditions it will take an adept in that practice to make it valuable, and it is not possible to bring it to the perfection one can hope to attain through a stethoscopic examination alone.—*Dominion Med. Monthly.*

---

### MEDICAL PEDAGOGICS.

A recent study on the subject of visceral anatomy by the author of "The Abdominal Brain" sums up the subject in the following conclusions: 1. Every college should have a chair of descriptive and applied visceral anatomy. 2. It should continue through the whole course of four years. 3. It should be taught by a practical abdominal surgeon. 4. Animal viscera should be employed the first year. 5. The chair should instruct every senior to perform experiments on the viscera of animals, and each student should perform a certain number himself, killing the animal and performing the autopsy. Such instruction will lessen the amateur and unnecessary abdominal surgery and the slaughter of the genitals which is so prevalent.

This is a fair sample of what is not infrequently urged as a part of the medical curriculum. At a time when medical studies have been lengthened so as to include a period of four years, and even with this length of time at their disposal, the framers of college curricula are at their wits ends to know what to include in the courses of study. Sound educators are generally of the opinion that six hours a day is about all that can be required, and at most this can cover but about nine or ten months in the year. Within this time must be crowded anatomy, physiology, medical chemistry, materia medica, therapeutics, embryology, surgery, obstetrics, and the practice of medicine. Not only must it include a large amount of practical work in laboratories and dissecting rooms, but we are now told that, in order to prevent "amateur and unnecessary abdominal surgery," we must establish a chair of descriptive and applied visceral anatomy, which shall continue through a whole course of four years. It is to be regretted that the author does not tell us exactly what is to be lopped off from the curriculum in order to provide a space for this new department of "applied visceral anatomy." It is believed by many that operations are often needlessly performed upon the skull for the treatment of epilepsy and other cerebral conditions which are not remediable by surgical intervention. Would not this be avoided by having a chair of applied cerebral anatomy, the course of instruction extending over the entire course of four years? We fail to see wherein the "convolutions of the abdominal brain" are more important than the convolutions of those contained within the cavity of the cranium. Improvement in the medical curriculum should come along the lines laid down by a late famous teacher, which is even more true than when it was uttered: "Surgery is studied by all and practiced by few; obstetrics is practiced by all and studied by few." There was never greater need of pedagogic science in our schools than now.

There is constant clamor for a so-called practical curriculum—one that teaches the manual dexterity and the application of science to the practice of medicine. If there were but few rules and a limited number of procedures, they could easily be included in the ordinary college course. The medical sciences have become so extended that only a limited portion of the field can be covered; hence it is of increasing importance that students should be thoroughly drilled in the basic principles of the science. The ideal medical curriculum of the future will include a teaching of anatomy, physiology, chemistry, and biology, apart from any suggestion of their application to the practical work of the physician in curing disease. With the principles of these sciences, taught by men who pursue them without thought of their application, we shall have students of medicine who will not need a special professor and four years of drilling to keep them from "the slaughter of the genitals."—*Medical Review.*

---

### OSTEOPATHS MUST LEAVE KENTUCKY.

Kentucky has once more rendered a service to humanity and the cause of legitimate medicine by pushing to successful conclusion an action in the courts relating to osteopathy. The facts are briefly as follows: Harry Nelson, an osteopath, applied for an injunction against the Kentucky State Board of Health, to stay proceedings against him instituted to prevent his practicing medicine in Kentucky. He claimed to be a graduate of the osteopathic institution of Kirksville, Mo., which he alleged was a reputable school.

The State Board of Health, having investigated the Kirksville institution by a special committee sent there for that purpose, denied that it was a reputable school of medicine. It appears that the authorities in control at Kirksville have erected a good building and expended large sums of money to foist their deceptive scheme upon an unwary public.

Judge Sterling B. Toney, before whom the case was tried, held that there were but two points at issue; first, the constitutionality of the act to protect citizens from empiricism; and, second, whether the Kirksville school was a reputable institution. He said the word reputable was not used here in its ordinary sense as when applied to individual character, but that it included a broader meaning as to the standing of the school among educational institutions, and whether it could register with the State Boards as a legally qualified medical school.

Judge Toney's opinion embraced a full review of the case and the essential points may be summarized as follows:

As to the first question, Judge Toney held that the act was constitutional quoting a number of State and federal decisions in support of his position. As to the second, whether or not the Missouri institution was a reputable medical college, one of good standing, he ruled that this was a question of fact to be decided by the State Board of Health: that that board had instituted a careful investigation of this matter, appointing a special committee to do this, and that as a result of this investigation it had decided that the Missouri School was not a reputable medical college, although legally chartered under the laws of that State. The board's decision on this question of fact Judge Toney held was final and conclusive, and was not a matter for judge or jury to pass upon. The injunction was refused and petition dismissed.

The State Board of Health regards the decision as effective in removing the last obstacle to freeing the State from the only remaining relic of quackery within its borders. It is to be regretted that the lawyers and courts of New York have not been as efficient in this work as they have been in Kentucky. Here it is difcult to inspire a lawyer with courage and enthusiasm sufficient to bring action, or to secure conviction when issue is made, or the courts to sentence when conviction is obtained. In Kentucky the bench and bar and all people of education are loyal to legitimate medicine, and desirous of maintaining its purity and high standards.—*Buffalo Medical Journal.*

---

(Abstract from the *Journal of the American Medical Association*, Sept. 9, 1899.)

### ACCIDENTAL WOUNDS OF THE FEMALE BLADDER.*

Accidental opening of the bladder has, for many years, been considered one of the most serious accidents that could occur in the course of the complicated work which gynecic surgeons are often called on to perform. The following case is offered in illustration of this type of injury:

M. H., unmarried, aged forty-one, was admitted to the City Hospital, Blackwell's Island, N. Y., September 30, 1898, suffering from a large myoma, which sprung from the anterior uterine wall and extended above the umbilicus. On October 3, the abdomen was opened, and the tumor, which weighed seventeen pounds, was drawn through an incision six inches in length, freed from its

*Presented by Frederick Holme Wiggin, New York City, to the Section on Obstetrics and Diseases of Women, at the Fifth Annual Meeting of the American Medical Association, held at Columbus, Ohio, June 6–9, 1899.

attachments and removed, together with the body of the uterus amputated near the internal os. As hemorrhage was profuse it became necessary to remove the mass very rapidly, to accomplish which the anterior attachment of the tumor was clamped and cut, when it was discovered, from the escape of urine, that the bladder had been opened near the fundus.

The general cavity had previously been shut off with gauze pads and thoroughly irrigated, followed by the use of Hydrozone in half strength, and this, in turn, by saline solution. The gauze pads were now changed, and the opening in the bladder, four inches in length, was closed by means of two layers of chromicized catgut sutures. The wound was then disinfected, and there being a large peritoneal flap, it was attached to the bladder and made to cover the line of sutures, thus making the bladder-wound extra-peritoneal. After further washing out of the abdominal cavity with Hydrozone and the saline solution the external wound was closed, without drainage, and the usual dressings applied. The patient being feeble it was not thought advisable to make a vesico-vaginal fistula to drain the bladder, but, instead, a self-retaining catheter was introduced. At the end of ten days, however, tumefaction occurred over the lower angle of the abdominal wound, and, on opening it, urine began to escape. A vesico-vaginal fistula was now made in order to afford adequate drainage. The sinus in the abdominal wall was curetted and, after being thoroughly disinfected with Hydrozone, its walls were sutured. Soon afterward, the sinus having closed, the sutures which kept open the vesico-vaginal fistula were removed, and the latter closed quickly without any further operative interference.

Percival (in *British Medical Journal*, 1897, vol. 1, p. 1282) reports a case of ruptured bladder on which he had operated. It was closed by means of a double wall of Lembert silk sutures. The wound in the abdominal wall was closed, after the peritoneal cavity had been flushed out with boric acid solution and a large quantity of clots and urinous fluids had been removed. For a few days the patient did well, and then died from peritonitis. But the necropsy proved that the bladder-wound had completely healed. It is the writer's opinion that had saline solution and Hydrozone been used, instead of boric acid, and the abdominal wound been closed leaving saline solution in the peritoneal cavity the patient would probably have recovered.

---

### NORDRACH AT HOME.

Physicians all over the world are now talking and writing about a noted Sanitarium in the Black Forest, Switzerland, for the special treatment of consumption, known as the Nordrach cure.

Here Dr. Walthers and his assistants carry out the treatment upon the modern ideas of rest, out of door life, proper feeding and required medication, and the results are wonderfully encouraging, between 70 and 80 per cent. of cures in cases not too long neglected.

What a contrast to the old methods of treatment, employed fifty years ago, when nearly every case of consumption ended sooner or later at the grave.

A physician who has lately spent some time in this Sanitarium studying these modern methods of treatment, says that wonderful results may be obtained at home with the Nordrach cure.

Proper exercise in a pure atmosphere, generous diet which should include regular doses of Scott's Emulsion of Cod Liver Oil, an out of door life, and plenty of sleep in rooms with the windows open, invariably bring about the desired result.

Too much reliance cannot be placed upon this Emulsion of Cod Liver Oil. It contains the best quality of oil in a finely emulsified condition; it does not separate, and as it is purely mechanical, no change takes place after bottling. It has great medicinal, as well as food value, as has been proven many times during the past quarter of a century.

Scott's Emulsion, pure air, rest and graduated exercise properly adjusted bring about a marked change; strength and vigor return, the tubercle bacilli are expelled, the flesh and appetite regained and health restored.

Try this treatment on your next case of consumption in the first stages.

### TREATMENT OF POSTERIOR URETHRITIS.

Dr. A. Ravogli, of Cincinnati, in the *International Journal of Surgery*, March, recapitulates the treatment as follows:

1. Irrigations with the Janet method in a recent case of gonorrhea will in many cases prevent posterior urethritis.

2. Irrigations with the recurrent catheter with permanganate of potassium, followed by injections of protargol, will cure in a relatively short time a case of subacute posterior urethritis without complications.

3. When chronic posterior urethritis lasting for a long time has caused infiltration of the submucous tissues, then the application of a sound with ichthyol salve gives the desired results.

---

### CHICKEN BROTH.

This is best when made from an old chicken. Wash and clean thoroughly, dissect joints, and chop all into small pieces, breaking the bones. Place it in a granite or porcelain-lined kettle. To one large chicken add one and one-half quarts of water, one teaspoonful of salt, and one tablespoonful of rice; let it simmer gently for two hours and reduce the quantity to one quart. Strain carefully and put to cool before serving, in order to remove all fat.—*Nursing World.*

---

### MORPHINE BEFORE ANESTHETICS.

About fifteen minutes before beginning a major operation (except in those necessitating an opening of the belly, in which cases the drug is highly objectionable) it is well to give one-quarter grain of morphine sulphate and one-hundredth grain atropine sulphate, hypodermically. Fully one half the chloroform will be saved, with corresponding diminution of danger.—*Louisville Medical Monthly.*

